# Supplementary material for: Bioinformatic Analysis of Sulfotransferases from an Unexplored Gut Microbe, Sutterella wadsworthensis 3_1_45B: Possible Roles towards Detoxification via Sulfonation by Members of the Human Gut Microbiome
Source: Int J Mol Sci. 2024 Mar 4;25(5):2983. doi: 10.3390/ijms25052983 (PMC10932419; doi:10.3390/ijms25052983)
Supplement: Supplementary file 1 [file ijms-25-02983-s001.zip › Figures S1-S8.pdf]

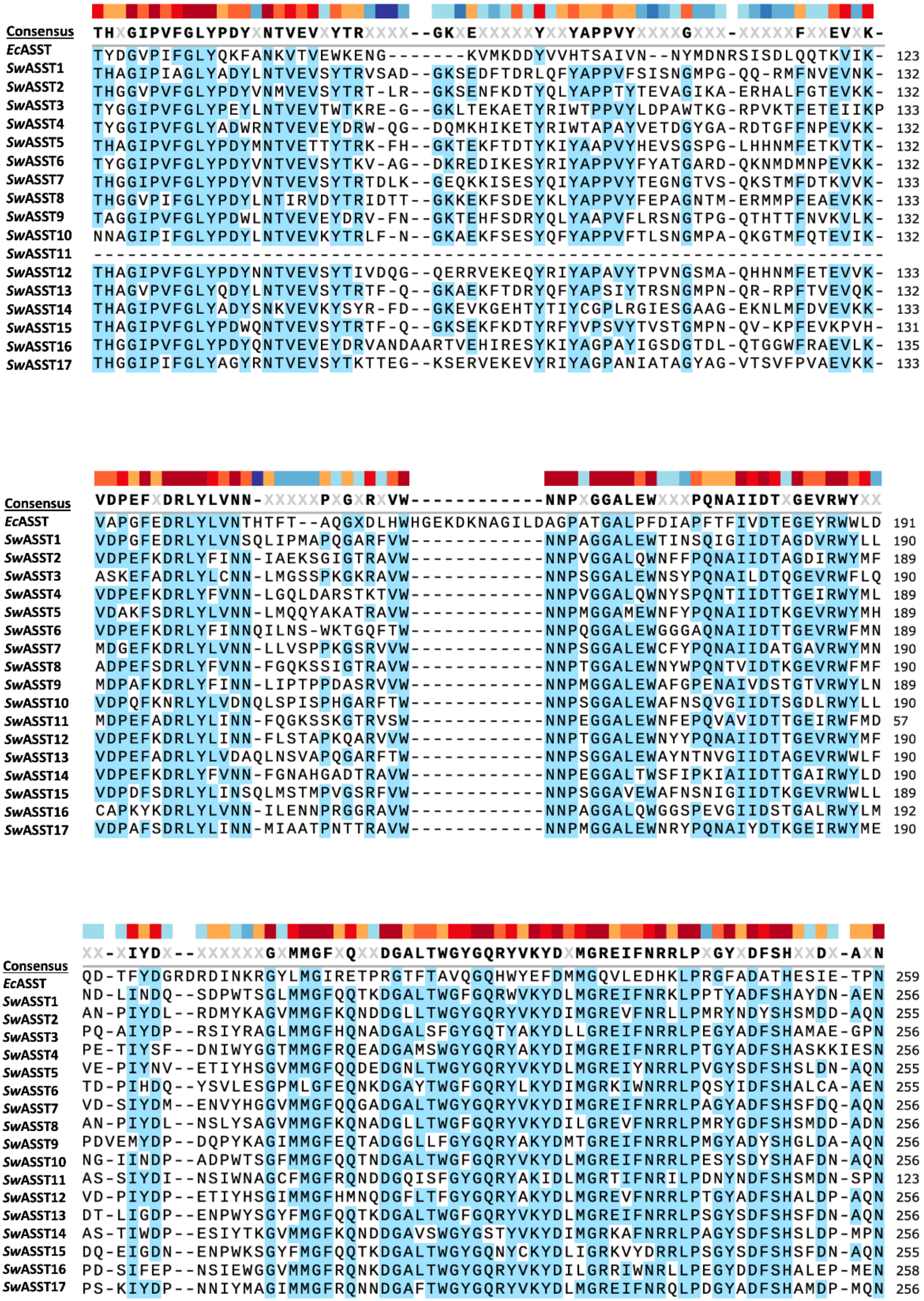

Figure S1. Partial multiple sequence alignment of all 17 *Sw*ASSTs with *Ec*ASST. Sequence alignment was performed on protein sequences without signal peptides. Color bar above aligned sequences shows the amino acid conservation at that position where dark red indicates highly conserved residues, while dark blue indicates little to no conservation.

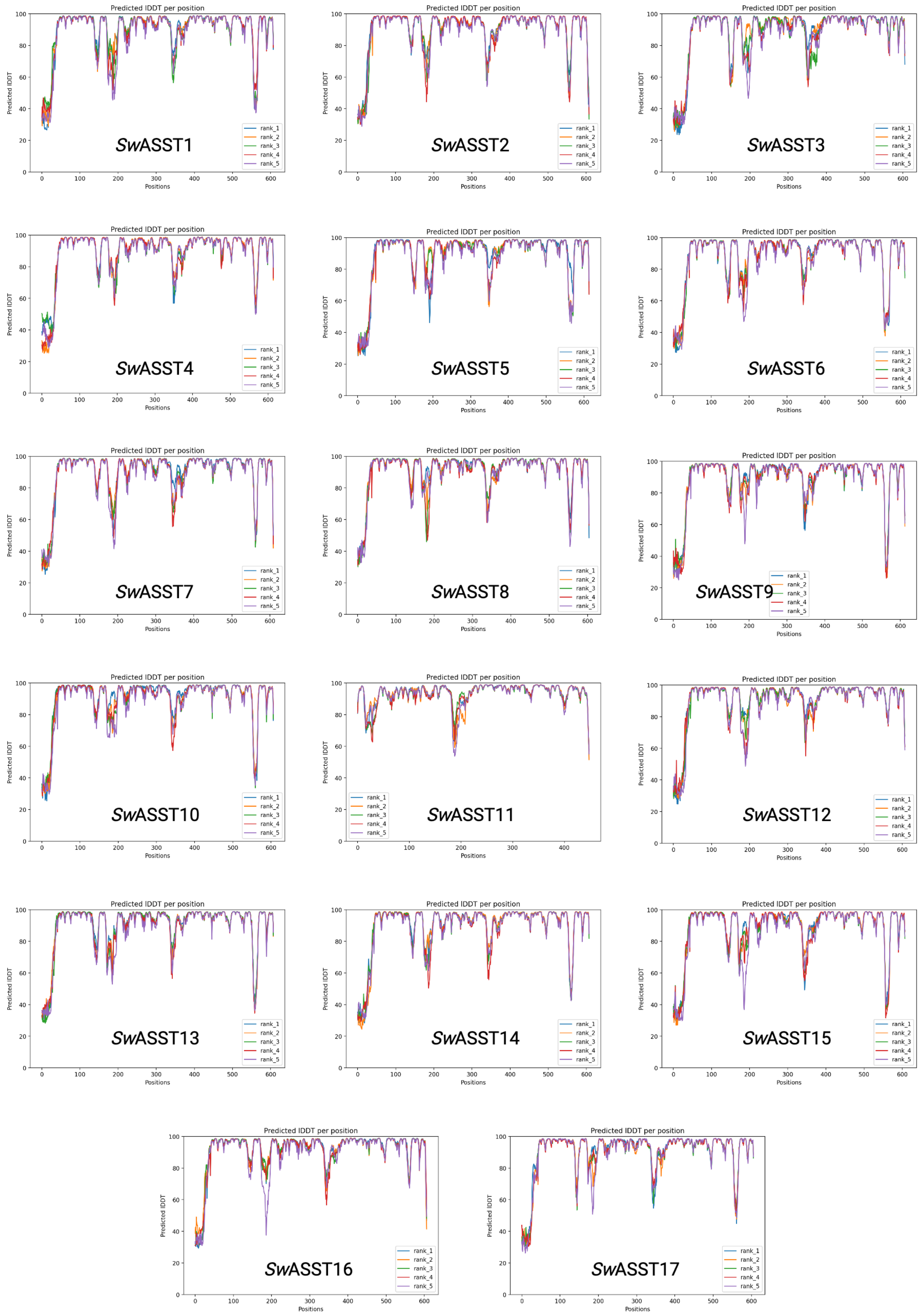

Figure S2. lDDT plots for all predicted structures of *Sw*ASST proteins generated by Alphafold2. Each plot has confidence percentage on the y-axis and the amino acid position on the X-axis.

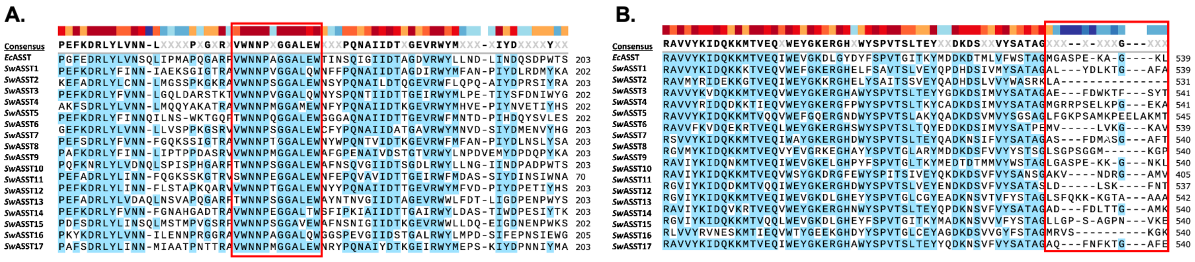

Figure S3. N and C terminus regions showing variable structural alignment. N and C terminus regions of *Sw*ASSTs that show high variations during structural alignments. A) Sequence alignment highlighting the N-terminus region that shows sequence conservation but structural variation. B) C-terminus multiple sequence alignment highlighting region with the red box that shows high secondary structure variability.

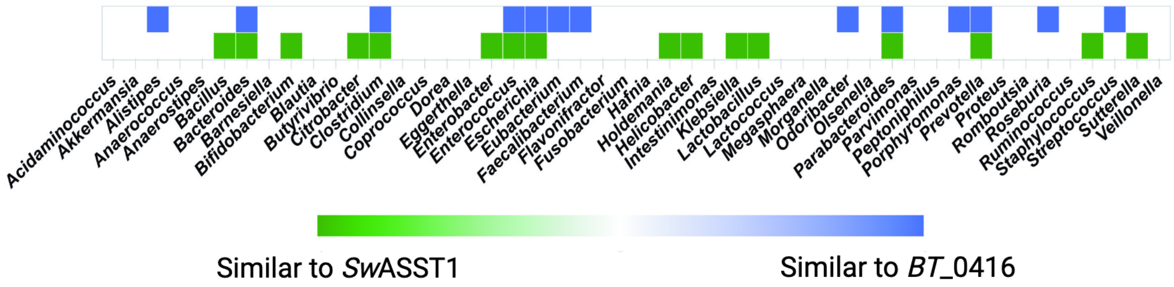

Figure S4. Distribution of PAPS-dependent and PAPS-independent sulfotransferases across members of the human gut microbiome. Heat map representation for the presence of sulfotransferase that are similar to *Sw*ASST1 (PAPS-independent) and/or *BT*_0416 (PAPS-dependent). Green boxes indicate the presence of sulfotransferases similar to *Sw*ASST1 whereas blue boxes indicate sulfotransferases similar to *BT*_0416.

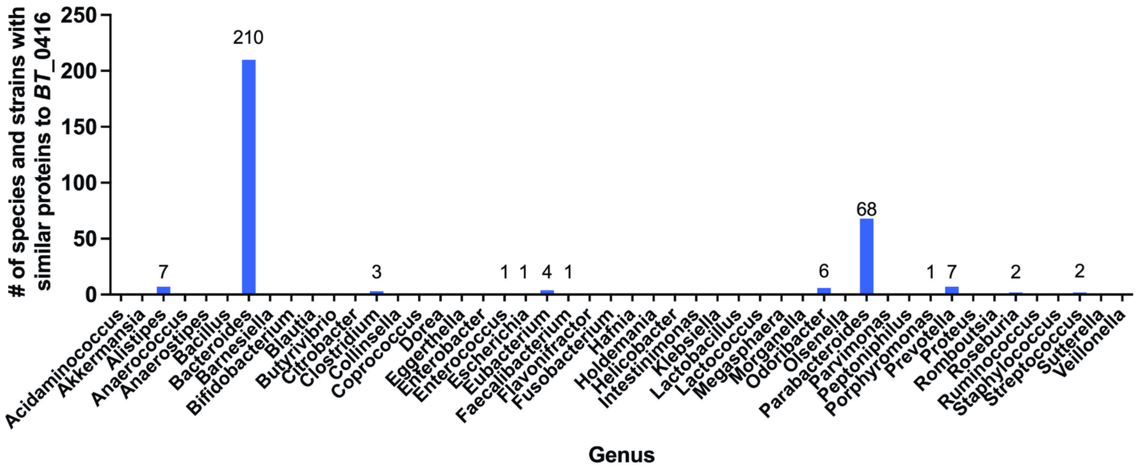

Figure S5. Human gut microbial genera with sulfotransferases similar to *BT*_0416. The bar graph represents total number of annotated sulfotransferases similar to *BT*_0416 in species and strains from each genus.

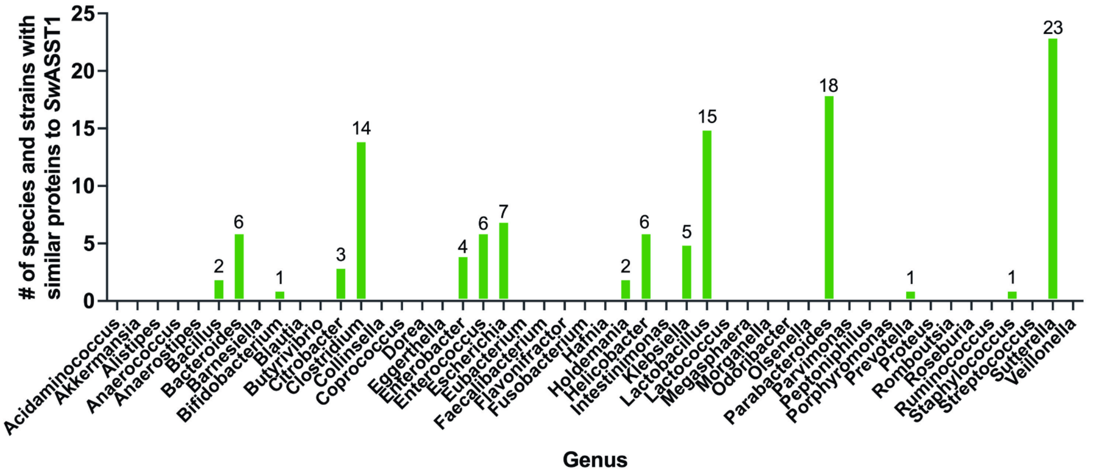

Figure S6. Human gut microbial genera with sulfotransferases similar to *Sw*ASST1. The bar graph represents total number of annotated sulfotransferases similar to *Sw*ASST1 in species and strains from each genus.

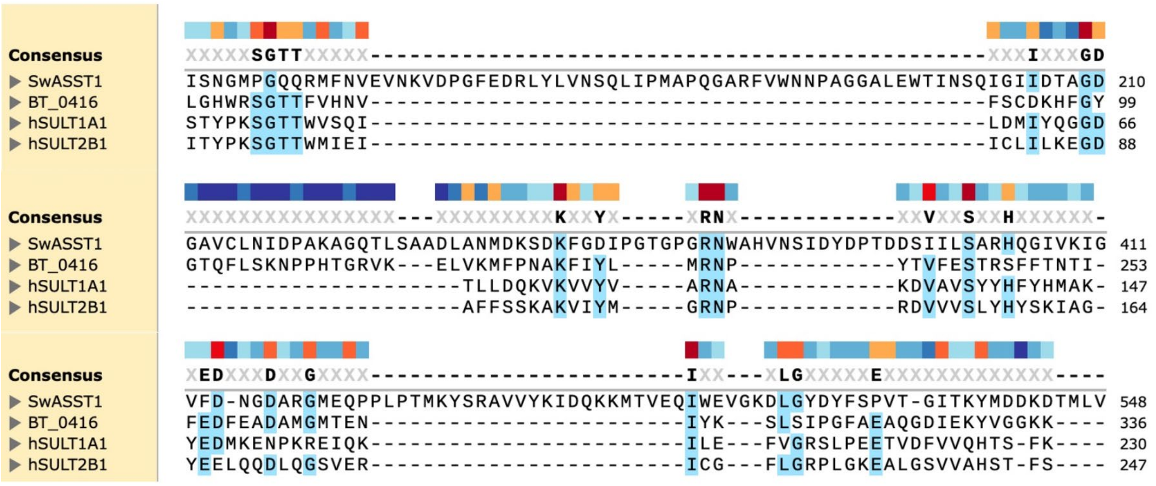

Figure S7. Multiple sequence alignment of *Sw*ASST1 with *Bacteroides BT*_0416, hSULT1A1, and hSULT2B1b. Light blue highlighted regions are well conserved regions and regions that share a high percentage of similarity. Color bar above the sequences show the amino acid conservation at that position, where dark red is the most conserved and dark blue is the least conserved position.

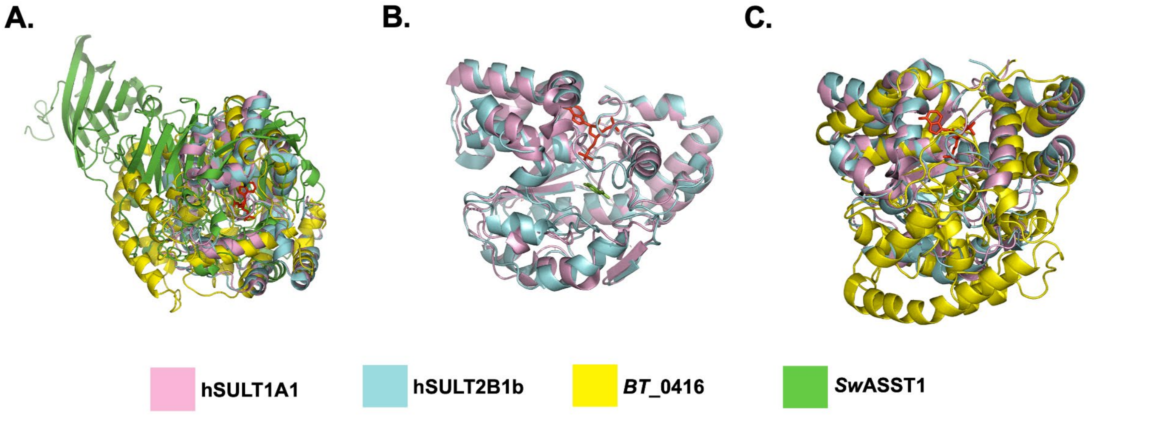

Figure S8. Structural alignments of *Sw*ASST1 with *BT*_0416, hSULT1A1, and hSULT2B1b. A) Alignment of all four sulfotransferases. B) Alignment of both human sulfotransferases, hSULT1A1 and hSULT2B1b. C) Alignment of three PAPS-dependent sulfotransferases (*BT*_0416, hSULT1A1, and hSULT2B1b). PAPS is shown in red and pNP is in lime green (visible in alignment B).
