# Supplementary material for: Bioinformatic Analysis of Sulfotransferases from an Unexplored Gut Microbe, Sutterella wadsworthensis 3_1_45B: Possible Roles towards Detoxification via Sulfonation by Members of the Human Gut Microbiome
Source: Int J Mol Sci. 2024 Mar 4;25(5):2983. doi: 10.3390/ijms25052983 (PMC10932419; doi:10.3390/ijms25052983)
Supplement: Supplementary file 1 [file ijms-25-02983-s001.zip › Table S1.pdf]

S1 Table. Human sulfotransferases and common substrates.

Protein	Length (#AAs)	Main Substrate/Compound Utilized	UniProt ID
SULT1A1	295	wide variety of acceptor molecules that have a hydroxyl or an amine group	P50225
SULT1A2	295	catecholamines, phenolic drugs and neurotransmitters	P50226
SULT1A3	295	phenolic monoamines, and phenolic and catechol drugs	P0DMM9
SULT1A4	295	phenolic monoamines, and phenolic and catechol drugs.	P0DMN0
SULT1B1	296	dopamine, small phenols, and thyroid hormones	O43704
SULT1C2	296	p-nitrophenol, and the carcinogenic N-Hydroxy-2-acetylaminofluorene	O00338
SULT1C3a	304	hydroxyl-chlorinated biphenyls	Q6IMI6
SULT1C3d	304	bile acids, thyroid hormones, and xenobiotic compounds	Q6IMI6
SULT1C4	302	phenolic compounds	O75897
SULT1E1	294	estradiol and estrone	P49888
SULT2A1	285	Hydroxysteroids and bile acids, known for selectivity of DHEA	Q06520
SULT2B1a	350	pregnenolone	
SULT2B1b	365	cholesterol	O00204
SULT4A1	284	Very low affinity for PAPS. Low catalytic activity towards common acceptors	Q9BR01

Shown are the amino acid length of each human sulfotransferase enzyme favored, along with favored substrates.
