# Supplementary material for: Bioinformatic Analysis of Sulfotransferases from an Unexplored Gut Microbe, Sutterella wadsworthensis 3_1_45B: Possible Roles towards Detoxification via Sulfonation by Members of the Human Gut Microbiome
Source: Int J Mol Sci. 2024 Mar 4;25(5):2983. doi: 10.3390/ijms25052983 (PMC10932419; doi:10.3390/ijms25052983)
Supplement: Supplementary file 1 [file ijms-25-02983-s001.zip › Table S2.pdf]

S2 Table. Annotated *asst* genes in the genus *Sutterella*.

***Sutterella* Species and Strain**	**# of Annotated *asst* Genes**
Sutterella wadsworthensis 3_1_45B	17
Sutterella wadsworthensis DSM 14016	15
Sutterella wadsworthensis HGA0222	15
Sutterella megalosphaeroides 6FBBBH3	11
Sutterella sp. KLE1602	11
Sutterella massiliensis An829	10
Sutterella seckii ASD393	10
Sutterella wadsworthensis 2_1_59BFAA	8
Sutterella faecalis KGMB03119	7
Sutterella seckii ASD3426	7
Sutterella sp. KCTC 15688	6
Sutterella parvirubra YIT 11816	5
Sutterella sp. AM11-39	2
Sutterella sp. AF15-44LB	1
Sutterella sp. AF15-45LB	1
Sutterella sp. AM18-8-1	1
Sutterella morbirenis	0
Sutterella sanguinus	0
Sutterella stercoricanis	0
Sutterella timonensis	0

Table depicts information shown in Figure 3. Number of annotated ASSTs for each species and strain are shown. Sutterella wadsworthensis 3_1_45B has the greatest with 17 ASSTs, while Sutterella morbirenis, Sutterella sanguinus, Sutterella stercoricanis, and Sutterella timonensis have zero.
