# Supplementary material for: Bioinformatic Analysis of Sulfotransferases from an Unexplored Gut Microbe, Sutterella wadsworthensis 3_1_45B: Possible Roles towards Detoxification via Sulfonation by Members of the Human Gut Microbiome
Source: Int J Mol Sci. 2024 Mar 4;25(5):2983. doi: 10.3390/ijms25052983 (PMC10932419; doi:10.3390/ijms25052983)
Supplement: Supplementary file 1 [file ijms-25-02983-s001.zip › Table S3.pdf]

S3 Table. ***S. wadsworthensis 3_1_45B* aryl-sulfate sulfotransferase information**.

Annotated Gene	Gene ID	Locus Tag	%GC	Predicted Protein	Length (AA)	Signal Peptide Length (AA)	Signal Peptide Type	Trans-membrane Region
*asst*1	650086806	HMPREF9464_00020	59	*Sw*ASST1	608	26	TAT	Yes
*asst*2	650089201	HMPREF9464_02348	61	*Sw*ASST2	609	22	Sec	No
*asst*3	650088873	HMPREF9464_02031	55	*Sw*ASST3	606	33	TAT	No
*asst*4	650088620	HMPREF9464_01783	55	*Sw*ASST4	615	32	Sec	Yes
*asst*5	650088457	HMPREF9464_01628	55	*Sw*ASST5	614	31	TAT	No
*asst*6	650088274	HMPREF9464_01448	49	*Sw*ASST6	612	25	TAT	No
*asst*7	650088224	HMPREF9464_01399	55	*Sw*ASST7	610	27	Sec	Yes
*asst*8	650087927	HMPREF9464_01116	54	*Sw*ASST8	605	20	Sec	Yes
*asst*9	650087562	HMPREF9464_00769	54	*Sw*ASST9	613	29	Sec	Yes
*asst*10	650087393	HMPREF9464_00601	50	*Sw*ASST10	607	24	Sec	No
*asst*11	650087285	HMPREF9464_00493	49	*Sw*ASST11	449	-	-	No
*asst*12	650086997	HMPREF9464_00211	57	*Sw*ASST12	608	28	Sec	Yes
*asst*13	650086849	HMPREF9464_00063	56	*Sw*ASST13	608	23	Sec	No
*asst*14	650087646	HMPREF9464_00851	58	*Sw*ASST14	608	25	TAT	Yes
*asst*15	650088356	HMPREF9464_01527	56	*Sw*ASST15	608	26	Sec	Yes
*asst*16	650086913	HMPREF9464_00127	60	*Sw*ASST16	607	23	TAT	No
*asst*17	650086936	HMPREF9464_00150	57	*Sw*ASST17	606	25	Sec	Yes

For each annotated gene, there is a gene ID, locus tag, and information that is presented visually earlier.
